# Editorial: Algal biomass and biofuels

**DOI:** 10.3389/fbioe.2022.1008760

**Published:** 2022-11-02

**Authors:** Kanhaiya Kumar, Wanthanee Khetkorn, Namita Khanna

**Affiliations:** ^1^ Fermentation and Microbial Biotechnology Division, CSIR–Indian Institute of Integrative Medicine (IIIM), Jammu, India; ^2^ Academy of Scientific and Innovative Research (AcSIR), CSIR–Human Resource Development Centre, Ghaziabad, India; ^3^ Division of Biology, Faculty of Science and Technology, Rajamangala University of Technology Thanyaburi, Thanyaburi, Pathumthani, Thailand; ^4^ Department of Biotechnology, Birla Institute of Technology and Science, Pilani, Dubai, United Arab Emirates

**Keywords:** photobioreactor, cultivation, spent media, fatty acids, secondary metabolites (SMs)

**FIGURE 1:**
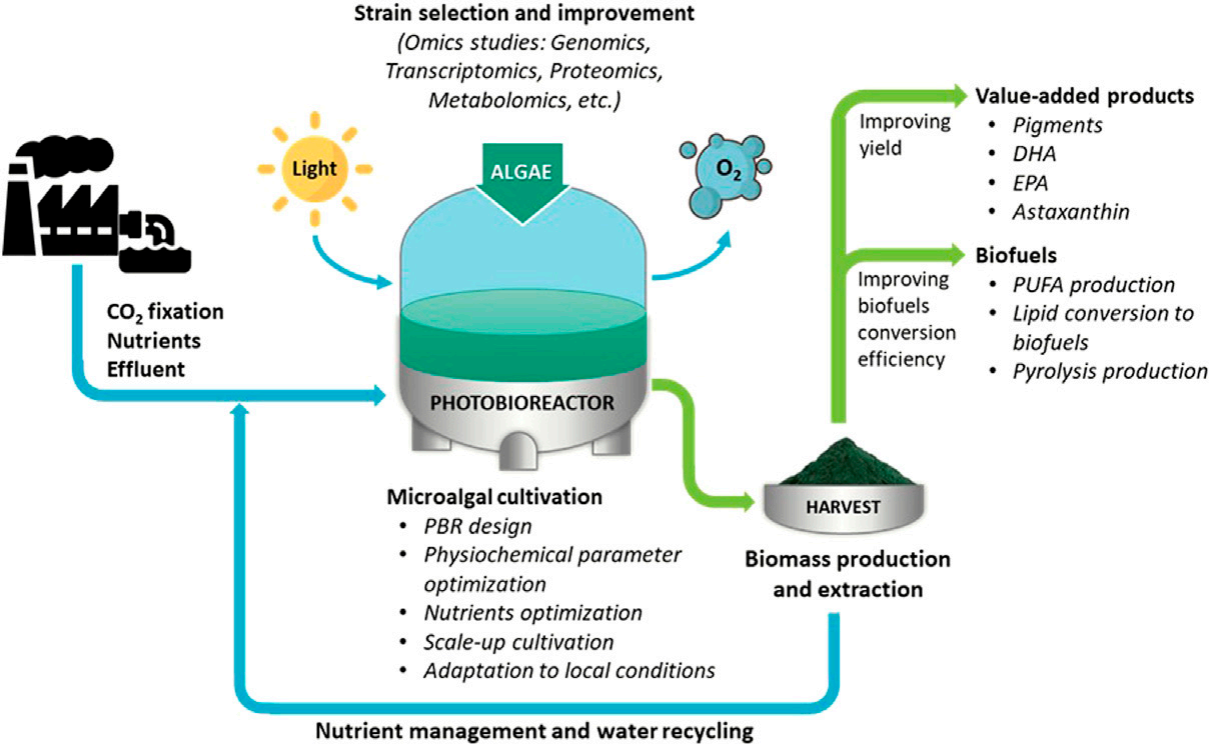
The challenges of algal technology for algal cultivation, biofuels production, and utilization of algal biomass to extract value-added products. PBR, photobioreactor; DHA, docosahexaenoic acid; EPA, eicosapentaenoic acid; PUFA, polyunsaturated fatty acids.

Algal technology is one of the promising areas of research that can alleviate several ongoing global challenges such as global warming, food, and fuel scarcity, and the growing demand for biopharmaceutical products for humans and animals. Algae bio sequester CO_2_ from the environment during photosynthesis and produce biomass. Algal biomass is a promising feedstock for extracting biofuels and commercially important biopharmaceutical products. The scope of this Research Topic is to collect high-quality research articles that can address the challenges associated with algal technology spanning over three broad domains: algal cultivation for biomass, biofuels production, and extraction of commercially valuable biomolecules (Figure 1). The process of algal biomass cultivation should be scalable, cost-effective, environment-friendly, and sustainable. The algal cultivation in photobioreactors has advantages over open ponds because of easy control of physio-chemical parameters and preventing contamination. In addition to these attributes, biomass should contain energy-dense or value-added biomolecules. The energy-dense biomolecules should be extracted and converted to biofuels efficiently, and the process should be benign to the environment. Several algal species are known to synthesize high-value secondary metabolites (SMs), such as docosahexaenoic acid (DHA), eicosapentaenoic acid (EPA), and astaxanthin. However, selecting the most promising strain and enhancing their yield is a top priority.

This Research Topic contains ten high-quality articles, which include nine original research articles and one review paper. Fifty two authors belonging to eight countries: India, China, Malaysia, Saudi Arabia, Australia, the United Kingdom, Belgium, and Canada, have participated in this Research Topic. Furthermore, some articles also show inter-country and inter-continental research collaborations.

The isolation of indigenous microalgal species, random mutation of them, and exploring psychrotolerant (low temperature) microalga for cold climate countries are some of the suggested strategies for sustainably improving the algal biomass productivity. Further, the algal cultivation process can be cost-effective and environment-friendly when applying a better nutrients management strategy and recycling spent water several times. Three original research articles are focused on microalgal cultivation for improving biomass production and CO_2_ fixation efficiency in a photobioreactor. Schoeters et al. cultivated the psychrotolerant snow alga *Chloromonas typhlos* at colder ambient temperatures in a 350 L pilot scale photobioreactor. Their primary purpose was to assess its biomass productivity and CO_2_ fixation efficiency. The study by Paquette et al. on cyanobacteria consortium at high pH and alkalinity revealed that spent cultivation medium could be reused at least five times by proper nutrient management without significantly inhibiting the biomass productivity. Farooq conducted a similar study of nutrient optimization (nitrogen and carbon dioxide) and water recycling during cultivation but used *Parachlorella kessleri* HY-6 as a model microalga. The author found the inhibitory effect of organics accumulated in the spent media for the subsequent cultivation and therefore applied activated carbon to remove the organics and improve the water recyclability. The water recycling strategy circumvents the water-intensive algal cultivation process, especially in arid countries.

Three original research articles describe the biofuels potential of microalgae. Jain et al. isolated several indigenous marine microalgae such as *Nannochloropsis oculate*, *Chlorella* sp., and *Planophila* sp., from the coast of the Arabian Sea. They explored their potential for polyunsaturated fatty acids (PUFA) synthesis, especially α-Linolenic acid. Similarly, Chen et al. isolated indigenous *Euglena gracilis* 815 and evaluated it as a new candidate for biodiesel production. The microalgal biomass of *Gonium pectoral* was a potential feedstock for pyrolysis. The study also assessed the CO_2_ biofixation potential along with pyrolytic kinetics of *G. pectoral* using kinetics modeling and an artificial neural network Altriki et al.

Four manuscripts containing three original research articles and one review article investigate the potential of microalgal biomass for secondary metabolites (SMs) production. The review summarized different genetic engineering studies targeting the improvement of microalgae for secondary metabolite production in microalgae (Sreenikethanam et al.). Three original research articles describe the production of SMs such as DHA, EPA, and astaxanthin from microalgae: *Schizochytrium* sp. (Zeng et al.), *Nannochloropsis oculata* (Razali et al.), and *Chromochloris zofingiensis* (Chen et al.), respectively. The mutagenesis on *Schizochytrium* sp. and *N. oculate* and nutrient optimization on *C. zofingiensis* enhanced the synthesis of SMs. The study on *Schizochytrium* sp., *N. oculata*, and *C. zofingiensis* was supported by metabolome, proteome, and bioprocess-based analysis, respectively. The atmospheric and room-temperature plasma (ARTP) mutagenesis combined with screening by iodoacetic acid and dehydroepiandrosterone enhanced the DHA synthesis in *Schizochytrium* sp. (Zeng et al.). The primary metabolites quantified using LC/MS correlated with the increased growth rate and DHA biosynthesis. The mutant strain produced the highest reported DHA concentration and productivity of 41.4 g L^−1^ and 430.7 mg L^−1^ h^−1^, respectively, at the end of 96 h fermentation. Similarly, the random mutagenesis coupled with the chemical (Ethyl methane sulfonate)-inhibitor-based selection method improved the EPA synthesis in the marine microalga, *N. oculata* (Razali et al.). The study also revealed the presence of alternative pathways for EPA synthesis when analyzed using label-free quantitative proteomics for differential protein expression. Based on Fatty acid methyl ester (FAME) data, the most suitable mutant strain had enhanced EPA content (1.7-fold) and EPA concentration (1.4-fold) compared to the wild type. Further, an effective two-stage heterotrophic cultivation of the unicellular green microalga, *C. zofingiensis*, enabled the synthesis of ultrahigh biomass and astaxanthin production (Chen et al.). *C. zofingiensis* was cultivated on a laboratory scale (7.5 L) and later scaled up in the 500 L fermenter. The combination of suitable concentrations of phytohormones gibberellic Acid-3 (GA3), C/N ratio, and NaCl enhanced the biomass and the astaxanthin synthesis. The highest biomass and astaxanthin yields were 235.4 g L^−1^ and 0.318 g L^−1^ (0.144% of DW), respectively. The astaxanthin yield was 5.4-fold more significant than the highest reported value in popular microalga, *Haematococcus pluvialis*. In this way, articles communicated in this Research Topic show significant improvement in the scientific knowledge base and contribute to the ongoing challenges of the commercialization of algal technology.

